# The Functionally Characterization of Putative Genes Involved in the Formation of Mannose in the Aplanospore Cell Wall of *Haematococcus pluvialis* (Volvocales, Chlorophyta)

**DOI:** 10.3390/metabo11110725

**Published:** 2021-10-23

**Authors:** Chunli Guo, Rui Mei, Muhammad Anwar, Di Zhao, Chengxiang Lan, Yanan Jiang, Jieyi Zhuang, Chaogang Wang, Zhangli Hu

**Affiliations:** 1 Guangdong Technology Research Center for Marine Algal Bioengineering, College of Life Sciences and Oceanography, Shenzhen University, Shenzhen 518055, China; guochunli@szu.edu.cn (C.G.); 2060251047@email.szu.edu.cn (R.M.); Anwar_uaar@yahoo.com (M.A.); dilysxiaodi@szu.edu.cn (D.Z.); 1900521008@email.szu.edu.cn (C.L.); 2060251001@email.szu.edu.cn (Y.J.); 1900251002@email.szu.edu.cn (J.Z.); 2 Key Laboratory of Optoelectronic Devices and Systems of Ministry of Education and Guangdong Province, College of Optoelectronic Engineering, Shenzhen University, Shenzhen 518060, China; 3 Shenzhen Engineering Laboratory for Marine Algal Biotechnology, Longhua Innovation Institute for Biotechnology, Shenzhen University, Shenzhen 518055, China; 4 Guangdong Provincial Key Laboratory for Plant Epigenetics, College of Life Sciences and Oceanography, Shenzhen University, Shenzhen 518055, China

**Keywords:** *Haematococcus pluvialis*, cell wall, mannose, genes, mechanism

## Abstract

Unicellular volvocalean green algal *Haematococcus pluvialis*, known as astaxanthin rich microalgae, transforms into aplanospore stage from the flagellate stage when exposed to the stress environments. However, the mechanism of the formation of aplanospore cell wall, which hinders the extraction of astaxanthin and the genetic manipulation is still unclear. In this study, the cell wall components under salicylic acid and high light stresses were explored, and cellulose was considered the main component in the flagellates, which changed gradually into mannose in the aplanospore stages. During the period, the genes related to the cellulose and mannose metabolisms were identified based on the RNA-seq data, which presented a similar expression pattern. The positive correlations were observed among these studied genes by Pearson Correlation (PC) analysis, indicating the coordination between pathways of cellulose and mannose metabolism. The study firstly explored the formation mechanism of aplanospore cell wall, which might be of scientific significance in the study of *H. pluvialis*.

## 1. Introduction

*Haematococcus Pluvialis* belongs to the genus *Haematococcus* of Chlorophyta, which enters gradually into static sporangium stage when exposed to high-intensity illumination, high osmotic pressure or nitrogen deficiency stresses. High content of astaxanthin (3,3′-dihydroxy ß-carotene-4,4′-dione) is enriched in this stage, which has been proved to be the biggest producer in nature [1]. Astaxanthin shows the strongest antioxidant activities compared with ß-Carotene, xanthophyll or other active substances [2,3,4,5,6,7]. However, *H. Pluvialis* has thick cell wall, which is responsible to hinders the extraction of astaxanthin. Both high content of astaxanthin and thickened cell wall were considered to respond to the stress environments [8,9]. Salicylic acid (SA) is a secondary metabolite, which is synthesized by a variety of prokaryotic and eukaryotic organisms, as a signal molecule of immune response in plants. SA plays a beneficial role in stress environments [10,11,12,13]. SA was proved to take part in the astaxanthin biosynthesis pathway and be utilized as a regulator for astaxanthin production in *H. pluvialis*. Higher astaxanthin productivity in *H. pluvialis* could be induced by SA [14,15,16,17,18,19]. SA might activate the formation of cell wall due to the simultaneous process with astaxanthin. However, the study conducted on metabolism of cell wall are still limited.

**Figure 1:**
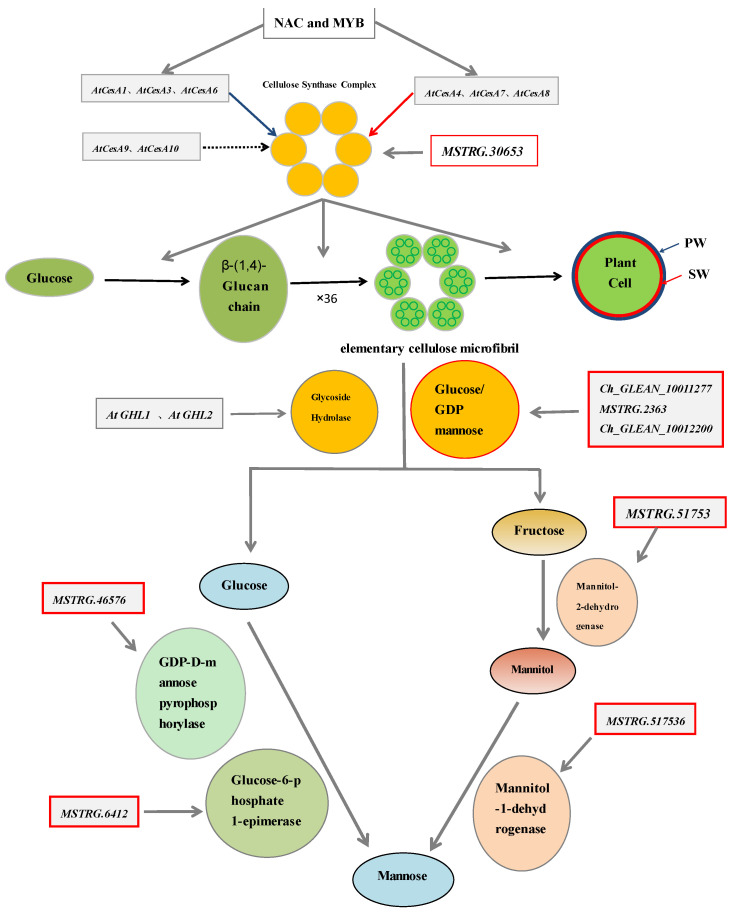
The putative metabolic pathways of mannose. Note: PW is the primary cell wall, SW is the secondary cell wall. Red arrow and the red cell wall means the cellulose in SW is regulated by these genes, and the blue arrow and the blue cell wall represent the cellulose in PW is generated by the corresponding genes. The genes in the red boxes are genes related to our study.

There is the extracellular matrix when *H. Pluvialis* existed in the green moving stage. During the process of entering into the sporangium stage, the primary wall, the triamine sheath and the secondary wall were successively formed beside the inner side of extracellular matrix. The primary wall and extracellular matrix disappeared subsequently, with the secondary wall left as the main structure of the thickened-cell wall [1]. Cyst cells stained with Calcofluor White and irradiated with UV light exhibited a continuous fluorescence in their walls. After being treated with cellulase, fluorescence decreased in both the primary cell wall and the secondary wall. The treatment of cysts with β-mannosidase decreased in the secondary wall and was discontinuous in the tertiary wall. The results revealed that both cellulose and mannose were proved to compose the secondary wall [9]. In higher plants, the cellulose is a glucan chain, which is synthesized by cellulose synthase (Ces A) [20]. It was one of the three components of the cell wall, including cellulose, hemicelluloses and lignin, that maintain the plant shape, provide rigid support and respond to the stresses [21,22]. Ces A belongs to the glycosyl transferase-2 (GT-2) superfamily with a conserved domain (pf03552). There are 10 member genes of Ces A family in *Arabidopsis thaliana*, among which AtCesA4, AtCesA7 and AtCesA8 are involved in the synthesis of the secondary cell wall, while AtCesA1, AtCesA3 and AtCesA6-like protein (AtCesA2, AtCesA5, AtCesA6 and AtCesA9) are mainly related to the synthesis of the cellulose in the primary cell wall [23,24,25]. The biosynthesis of cellulose could be initiated by the multi-level transcriptional regulatory network composed of NAC and MYB main transcriptional factors switches and their downstream target genes [26]. Enzymatic hydrolysis of cellulose in the organisms is accomplished by cellulase, which is composed of exoglucanase, endoglucanase and glucosidase [27]. The previous study indicated that the breaking of β-1,4 glycosidic bond led to form glucose carboxylate and then monosaccharide under the enzymatic hydrolysis (Figure 1) [28].

It has been observed that the fructose was dehydrogenated to form mannitol, which is catalyzed by mannitol-2-dehydrogenase, while mannitol to form mannitol by mannitol-1-dehydrogenase [29,30]. In previous study, it was reported that mannitol-2-dehydrogenase and formate dehydrogenase could catalyse fructose to generate mannitol, and mannitol-1-dehydrogenase coupled with NADH oxidase to catalyse mannitol dehydrogenation to form mannitol (Figure 1) [31].

In previous study, we observed that both the astaxanthin synthesis and the cyst cell wall formation in *H. pluvialis* when exposed to the treatment of salicylic acid (SA) and hight light (HL) were faster than the treatment of HL or SA. Hence, an exploration of the molecular mechanism of *H. pluvialis* in the regulation of the formation of cell wall upon exposure to SA and HL stresses is evaluated by transcriptomic analysis. The components of the cyst cell wall were subsequently detected, proved mannose to be the predominant component. In order to determine the formation mechanism of mannose during the period into the cyst cells, the comparative expression profiling under the stress of SA and HL were studied. Here, we reported the genes, which are regulating the development of both cellulose and mannose during the process of entering sporangium stage. Finally, the hypothesis of the formation pathway of mannose in *H. Pluvialis* was put forward that in appropriate growth conditions, cellulose synthetase is utilized to form cellulose. However, when exposed to SA and HL stresses, cellulose hydrolase is upregulated to form fructose and glucose and then finally transforms into mannose under the catalysis of mannitol dehydrogenase and mannose isomerase, respectively. The results in this study provided valuable information to illustrate the molecular mechanisms of coordinate relations between cellulose and mannose biosynthesis. The formation of mannose is a self-protection mechanism that might help algal cells to survive the under-stress environment or be beneficial to the existence of astaxanthin, helping the adaption of *H. pluvialis* to stress conditions.

## 2. Results

### 2.1. Morphological Changes of Algal Cells in Responding to SAHL Stresses

**Figure 2:**
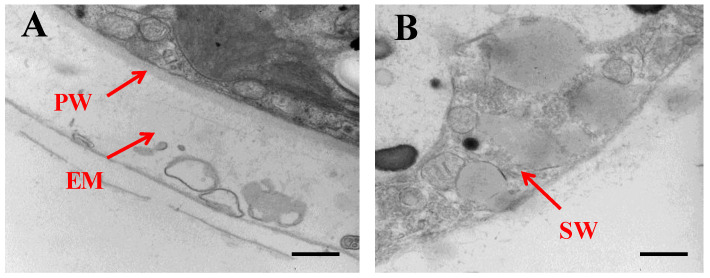
The morphology of cell wall of *H. pluvialis* in the aplanospore stage under the stress of SAHL. (**A**) The cell wall of *H. pluvialis* under the normal condition for 48 h as control. (**B**) The cell wall of *H. pluvialis* in SAHL_48 stage. PW and EM represent primary wall and extracellular matrix, SW in picture B is the secondary wall. Bars represent 500 nm.

The morphology of cell wall in the aplanospore stage observed by TEM. The extracellular matrix and the primary cell wall disappeared, with the thickened secondary cell wall left as the primary structure (Figure 2).

**Figure 3:**
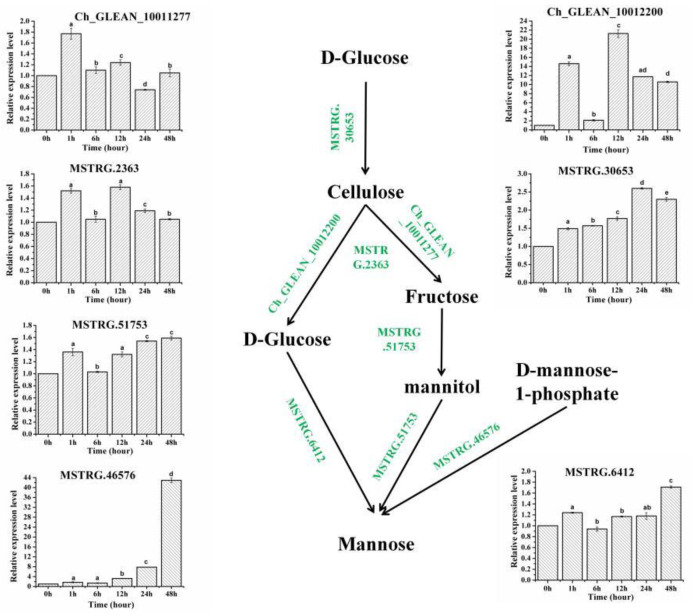
Expression of cellulose and mannose biosynthesis-related genes in *H. pluvialis* under different treatment stages. Note: Different superscript lowercase letters on top of the columns indicate significant differences (*p* < 0.05) among the treatments, and any of the same lowercase letters or letter said the difference was not significant (*p* > 0.05).

The contents of the cellulose and lignin in *H. pluvialis* cells harvested from different times stressed by SAHL were measured. The pattern of the batch growth is measured in Table 1. All samples began with a cell density of 9.92 ± 0.91 × 10^4^ cell·mL^−1^. We focused on the component of cyst cell wall in SAHL_48 stage because the cyst cells were formed in SAHL_48 and they were clearly observed, which helped to study the contents of cyst cell wall in detail. During the process from stage control to SAHL_48, the contents of cellulose and mannose components of cell wall were increased along with time course of SAHL treatment, and the contents of all the components in SAHL_48 arrived most which proved the mannose to be the main structure of cyst cell wall in stage SAHL_48 (Table 1). According to the results of the investigation, the contents of all the observed components in stage SAHL_1 showed drastic increase, which might be due to the upregulated expression of the corresponding genes (Figure 3). The cellulose contents were observed about 388.49 ± 4.67 ng/g in SAHL_48 treatment, which were higher than control (300.16 ± 2.74 ng/g). The mannose contents in SAHL treatment were measured at 293.89 ± 1.58 µg/g in SAHL_48, higher than that of control 180.13 ± 2.12 µg/g. The results showed that the mannose contents were remarkably higher in response to the stress of SAHL. We have found from these results, that mannose proved to be the main component of cell wall in the aplanospore stage when the algae cells were exposed to the SAHL treatments. The contents of fructose and glucose were increased from 0.83 ± 0.01 mg/g and 0.76 ± 0.01 mg/g to 1.33 ± 0.01 mg/g and 1.16 ± 0.01 mg/g respectively, during the transit stage process from flagellum to the aplanospore stage.

### 2.2. The Genes Involved in the Development of the Cell Wall of H. pluvialis When Exposed to the SAHL Stresses

**Figure 4:**
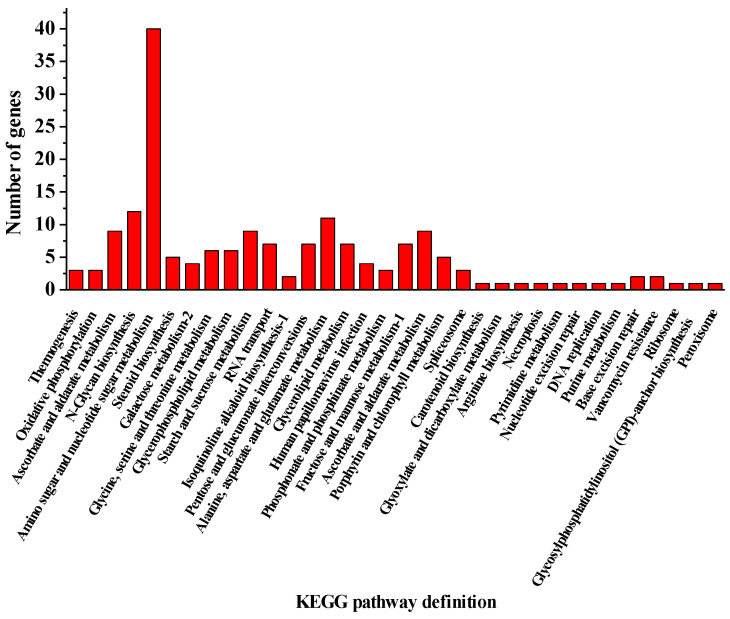
KEGG pathway classification of the unigenes involved in the development of cell wall. The X-axis presents specific pathways in the third hierarchy.

**Figure 5:**
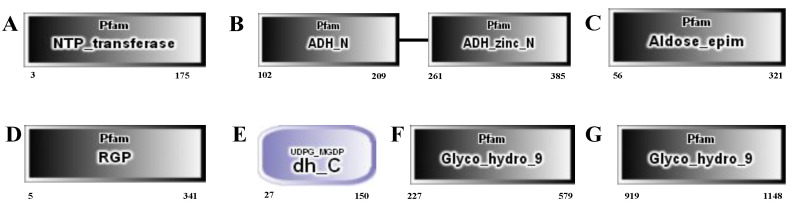
The functional domains of the putative cell wall genes. (**A**) The NTP transferase functional domain of MSTRG.46576; (**B**) The catalytic domain of alcohol dehydrogenases functional domain of MSTRG.51753; (**C**) The Aldose 1-epimerase functional domain of MSTRG.6412; (**D**) The UDP-arabinopyranose mutases and putative alpha-1,4-glucan-protein synthase functional domains of MSTRG.30653; (**E**) The UDP-glucose/GDP-mannose dehydrogenases functional domain of MSTRG.2363; (**F**) The O-Glycosyl hydrolases functional domain of Ch_GLEAN_10012200; (**G**) The O-Glycosyl hydrolases functional domain of Ch_GLEAN_10011277.

The analysis of the transcriptomic effect of salicylic acid and high light on lipid and astaxanthin accumulation in *H. pluvialis* was formerly conducted [32]. Based on the NCBI database numbered PRJNA675306, the comparative transcriptome analysis of *H. pluvialis* among the treatment stages highlighted 348 unigenes to be putative cell wall, membrane or envelope biogenesis related genes which were in response to SAHL stresses according to the KEGG pathway. Among the character differently expressed cell wall genes, up to 34 KEGG pathways were identified (Figure 4). The mostly genes involved in the metabolic pathways were amino sugar and nucleotide sugar metabolism (40 genes), N-Glycan biosynthesis (9 genes), pentose and glucuronate interconversions (7 genes), alanine, aspartate and glutamate metabolism (11 genes), starch and sucrose metabolism (9 genes), fructose and mannose metabolism-1 (7 genes), and ascorbate and aldarate metabolism (9 genes), and so on. The genes related to the pathways of N-Glycan biosynthesis, pentose and glucuronate interconversions, starch and sucrose metabolism and fructose and mannose metabolism-1 were coincident with the development of carbon metabolisms (Table 2). Then both the key cellulose and the mannose related genes were sequenced, and the Open Reading Frame (ORF) and putative protein sequences were then obtained by using the software DNASTAR Lasergene EditSeq (V 7.1.0) (http://50328.xc.zhongguohao123.com/down/dnastar%20lasergene%E7%A0%B4%E8%A7%A3%E7%89%88%20V7.1.0@1166_4076.exe (accessed on 4 March 2018). The functional domains of the related genes were explored according to the functional domains predicted with SMART (V 9) (http://smart.embl-heidelberg.de/smart/set_mode.cgi?NORMAL=1 (accessed on 26 October 2020) based on the transcriptome sequences (Figure 5).

### 2.3. The Expression Profiles of Genes Involved in the Biosynthesis Pathways of Cell Wall

In this study, the expression of both key cellulose and mannose development genes were determined upon different SAHL treatment stages. The expression level of these genes varied significantly among the stages (Figure 3). Among them, three putative genes were predicted to encode O-Glycosyl hydrolases and Glucose/GDP mannose dehydrogenase, which endowed the proteins the property of cellulose [33,34], and the gene_id were Ch_GLEAN_10011277, MSTRG.2363 and Ch_GLEAN_10012200, respectively. The cellulose genes showed the same trends that the genes were significantly upregulated when exposed to the SAHL treatment for 1 h and 12 h. One gene MSTRG.30653 encoding UDP-arabinopyranose mutase was considered to play an important role in the biosynthesis of cellulose [35,36,37], which turned to be upregulated in the different treatments. MSTRG.6412, MSTRG.51753 and MSTRG.46576 were explored to encode Glucose-6-phosphate 1-epimerase, mannitol dehydrogenase and GDP-D-mannose pyrophosphorylase, respectively, which were confirmed to take part in the biosynthesis of mannose [29,30,31,38,39,40]. The mannose gene MSTRG.46576 showed the significant difference when stressed by SAHL treatment for 48 h. Meanwhile, the genes MSTRG.6412 and MSTRG.51753 shared the same expression trends among the different treatment groups, which displayed upregulated when treated for 1 h, and down-regulated subsequently when stimulated to 6 h. The expression levels were increased as the treatment continued for 48 h. According to differential numbers of the unique transcripts, the potential regulatory contributions of these cell wall-genes might be characterized differently in the cellulose biosynthesis and hydrolysis pathways, and also the mannose biosynthesis pathways of this green microalgae.

### 2.4. Correlations between Expression of the Cell Wall Genes and the Components

In this study, expressions of both the key cellulose and mannose biosynthesis genes during the SAHL stresses were confirmed. To explore the relationship of these predicted genes with the cellulose and mannose, Pearson Correlation (PC) analysis (SPSS 19.0) was carried out, and the results were displayed in Table 3. Differences in the correlations were tested between the genes and components. As we could see, none of the gene expressions were observed negatively correlated with the components except Ch_GLEAN_10011277. The MC shared close correlations with that of the mannose biosynthesis genes MSTRG.6412, MSTRG.51753 and MSTRG.46576 were analyzed in this study, while it was significantly correlated with the gene MSTRG.3065 involved in cellulose biosynthesis pathway. The CC was perceived significantly correlated with the cellulolytic enzyme Ch_GLEAN_10012200 and MSTRG.2363, and also with the cellulose synthase MSTRG.30653. The CC was also predicted to show a close relationship with MSTRG.51753, a gene plays an important role in the mannose biosynthesis pathway. In consideration of glucose and fructose as the resources of mannose, we conducted the relationship of FC and GC with the putative genes in this study. It was observed that FC significantly correlated with cellulose synthase MSTRG.30653 as well as with the mannose biosynthesis genes, such as MSTRG.51753 and MSTRG.6412. The GC was found to have close relationships with MSTRG.30653 and MSTRG.6412 (Table 3).

## 3. Discussion

As is well known that the cell wall of *H. pluvialis* in the cyst stage greatly hinders the extraction of astaxanthin, and might be the biggest obstacle to the genetic manipulation. Great efforts by biochemical analysis method had been made to explore the components of the cell wall of *H. pluvialis* in the different development stages. Cyst cells stained with Calcofluor White and irradiated with UV light exhibited a continuous fluorescence in their walls. After being treated with cellulase, fluorescence decreased in both the primary cell wall and the secondary wall. The treatment of cysts with β-mannosidase decreased in the secondary wall and was discontinuous in the tertiary wall. The results revealed that the cell wall in the cyst stage was mainly composed of mannose besides cellulose [1,9]. However, the results of our study declare that during the period into the cyst stage, both the cellulose and mannose increased as the main components of cell wall, which is consistent with the previous papers reported, the mannose played the role as the main component compared with cellulose. Additionally, lignin is also detected as fewest component, which is not mentioned in our paper.

According to the study, the genes involved in the hydrolysis pathways of the cellulose showed doublet trend, which reaches the highest value in SAHL_1 and SAHL_12, respectively. The gene played an important role in the biosynthesis pathway of cellulose presented gradually upregulated until SAHL_24, and then reduced. Based on the characterized results, we speculated that when the SAHL treatment is prolonged, the monosaccharide products of hydrolysis might provide substrates for the resources of mannose and astaxanthin [29,31]. The synthesis of cellulose in the SAHL_24 stage might improve the rigid support of mannose to construct the cell wall [1,9]. We declared that the processes of both the biosynthesis and hydrolysis of cellulose might remain in a state of dynamic balance that the cellulose is hydrolyzed as it is synthesized.

The previous studies reported indicate that all of the cellulase genes, which are identified in the higher plants were designated as glycosyl hydrolase family 9 (GH9) [33,34]. GH9 is considered to be one of the most extensive types of cellulases. These enzymes are widely distributed in plants, animals, bacteria and fungi species. Enzymes that contain the GH9 catalytic domain can have at least four distinct molecular architectures [41]. The enzymatic hydrolysis of cellulose is accomplished by cellulase in organisms. Cellulase is a kind of complex enzyme, which is composed of three enzymes, such as exoglucanase, endoglucanase and glucosidase [27]. The cellulase that is identified in our study, belong to the endoglucanase. According to the co-relationship analysis, the gene Ch_GLEAN_10012200 encoding O-Glycosyl hydrolases and the gene MSTRG.2363 encoding Glucose/GDP mannose dehydrogenase have a significantly close relationship with the cellulose in functionally characterized different treatments. However, Ch_GLEAN_10011277, is another predicted gene encoding O-Glycosyl hydrolases, has no significant close relationship with the cellulose, which needs further identification and confirmation. Above among all mentioned genes, our study showed that Ch_GLEAN_10012200 and MSTRG.2363 genes might play an important role in the development of cellulose and in the morphology reconstruction in respond to the SAHL stresses.

*H.pluvialis* is the special green algae that the cell wall is mainly comprised of mannose when exposed it to the environment stressed compared with other plants. However, the mechanism associated with development of mannose in *H.* *pluvialis* was seldomly studied. GDP-D-mannose pyrophosphorylase is a key enzyme in L-galactose synthesis pathway, which catalyzes the formation of GDP-D-mannose from D-mannose-1-phosphate. GDP-D-mannose is not only a precursor of VC synthesis but also involved in cell wall polysaccharide synthesis and protein glycosylation. GMP gene has been cloned from *Arabidopsis*, peach and tomato [38,39,40]. In Dendrobium officinale, GDP-mannose pyrophosphorylase is involved in the pathways of synthesis of glucomannan, ascorbic acid and glycosylation, which plays an important role in the normal growth and provide resistance against stress in plants [2,3,42,43]. The enzyme has been successfully isolated and purified from higher plants such as *Arabidopsis* thaliana. The full length of the GDP mannose pyrophosphorylase gene and its function were previously studied [44].

In recent study, it was observed that the GDP-D-mannose pyrophosphorylase gene MSTRG.46576 was significantly upregulated during the period of SAHL treatments, and the highest expression level was obtained at the SAHL_48 stage. Additionally, the Pearson Correlation (PC) analysis (SPSS 19.0) revealed that MSTRG.46576 had a close relationship with the change of mannose. Based on the results of these studies, we came to the conclusion that the gene MSTRG.46576 encoding GDP-D-mannose pyrophosphorylase might take participate in regulating the synthesis of mannose and the re-construction of the cell wall upon exposure to the SAHL stresses.

Cellobiose 2-epimerase was cloned and expressed in *E. coli* which catalyzed the epimerization of aldose with C2 right configuration and C3 left configuration, and isomerizes D-glucose to form D-mannose. L-RHA isomerase (Rha A) of Thermotoga maritima could transform fructose into L-mannose. D-fructose can be transformed into D-mannose by the recombinant D-source sugar isomerase from Providencia stuartii [45,46,47]. Mannitol-1-dehydrogenase is found in celery, which can catalyze the conversion of mannitol to mannose under the action of NAD^+^ [29]. Mannitol, the substrate of mannose synthesis, can be produced by fructose under the joint action of mannitol-2-dehydrogenase and coenzyme NADH or NADPH [31].

The genes including MSTRG.6412 and MSTRG.51753 identified in this study encode glucose-6-phosphate 1-epimerase and mannitol dehydrogenase, respectively. In consideration that the fructose and glucose might be the substrates of mannose under the characterized catalysis of mannitol dehydrogenase and glucose-6-phosphate 1-epimerase, we detected the specific components content in the different treatments. The results predicted that both the fructose and glucose increased as the treatments prolonged, as well as the key component cellulose. The results obtained from this study indicated that the contents of glucose and fructose as well as the key components of cellulose showed increasing trend, when treatments were prolonged. Both MSTRG.51753 and MSTRG.51753 increased during the development of the cell wall. Finally, the Pearson Correlation (PC) analysis (SPSS 19.0) revealed that both the gene MSTRG.6412 and MSTRG.51753 represented a significant relationship with the mannose. We then declare that the gene MSTRG.6412 and MSTRG.51753 encoding glucose-6-phosphate 1-epimerase and mannitol dehydrogenase might play important roles in the synthetic pathway of mannose (Figure 1).

## 4. Materials and Methods

### 4.1. H. pluvialis Strain and Culture Conditions

*H. pluvialis* strain 192.80 was used in this study. It was preserved in our laboratory, which was originally purchased from EPSAG (Experimental Phycology and Culture Collection of Algae, Goettingen University, Goettingen, Germany). The algal cells were kept in especially soil phosphoric (ESP) medium at 22 °C with continuous illumination of 25 µmol photon m^−2^·s^−1^ in a growth chamber till to the logarithmic phase (about 1 × 10^5^ cells·mL^−1^). The algae cells were then transferred to the stress of salicylic acid (SA) and high light (HL, 350 μmol·m^−2^·s^−1^) treatments (SAHL), and the SA dosages were assigned at 25 mg·L^−1^ [18,19]. The cultures were kept for 0 h, 1 h, 6 h, 12 h, 24 h and 48 h, which were assigned as Control, SAHL_1, SAHL_6, SAHL_12, SAHL_24 and SAHL_48, respectively. The samples were then harvested at 6000× *g* for 10 min, frozen in liquid nitrogen immediately and stored at −80 °C for further analysis. At least three biological replications were performed for each testing sample.

### 4.2. Observation of Microalgal Morphology by Transmission Electron Microscope (TEM)

Algal cells in SAHL_ 48 stage were collected under the condition of 1600× *g* for 10 min. The stationary solution was prepared by dissolving Glutaraldehyde/paraformaldehyde in dimethyl arsenate buffer (0.05 M; pH 7.2). The cells were immobilized in dimethyl arsenate buffer containing 2% tetraoxide (*w*/*v*) and uranium acetate solution (2% saturated aqueous solution, pH 4.5) overnight. Dried in methanol and embedded in EMS medium. The condition of depressurization should be operated for the final concentration of algal cells. Stained with 2% uranium acetate saturated aqueous solution for 30 min and lemon lead for 12 min following the section. Finally, the microalgal morphology was observed under the transmission electron microscope (No: H-7650, Hitachi, Japan).

### 4.3. The Measurement of the Components Comprised Cell Wall

In order to determine the components of the cell wall upon exposure to the stress of SAHL, the algae cells in the logarithmic growth phase were transferred to SA and high intensity of illumination for 0 h, 1 h, 6 h, 12 h, 24 h and 48 h, respectively. The cells under the normal intensity of illumination from the corresponding time were sampled as control. The cells were harvested by centrifugation at 4000 rpm for 5 min and frozen by liquid nitrogen immediately. The whole cells were utilized to determine the change of cellulose, mannose and their intermediate metabolites due to their uniqueness to the cell wall by Qingdao Science Innovation Quality Testing Co., Ltd. Company (Shandong, China). Three biological replications were carried out for each testing sample.

#### 4.3.1. The Measurement of Cellulose

The content of cellulose was detected using Enzyme Linked Immunosorbent Assay For Plant Cellulose Test Kit (No: AB-C11602B, Kete Biotechnology, China). The algal cells were ground into powder. The standard cellulose was diluted to 25, 50, 100, 200 and 400 ng/L to obtain the standard curve. The plate was subsequently incubated at 37 °C for 30 min after being sealed with sealing film. The concentrated washing solution was diluted with 30 times deionized water. The sealing film was then carefully removed to make sure the plate was free from liquid. Each well was filled with diluted washing solution and left standing for 30 s, which was repeated 5 times. 50 μL enzyme labelled reagent was added to each well except the blank well. After being incubated at 37 °C for 30 min, each well was filled with washing solution, stood for 30 s after the solution was discarded, repeated for 5 times. Fifty microliters of both developers A50 and B50 were added to each well, and the color was developed at 37 °C in the dark for 10 min after being mixed gently. 50 μL termination solution was added to each well to terminate the reaction (at this time, the blue color turned to yellow). The absorbance of each well was measured at 450 nm.

#### 4.3.2. The Measurement of Mannose, Glucose and Fructose

The content of mannose in cyst phase was detected by Mannose Content Detection Kit (No: SEB542Hu, Kete Biotechnology, China). The standard mannose was dried at 96 °C ± 2 °C for 2 h and then dissolved with deionized water. Five milliliters hydrochloric acid was then added, and the mannose and hydrochloric acid solution were diluted to 1 L with deionized water to prepare mannose standard solution.

The sky-blue copper hydroxide precipitate was formed immediately after the same amount of basic copper tartrate A and B were mixed. The precipitate reacted with the sodium tartrate quickly to achieve a dark blue soluble potassium sodium tartrate copper complex. Under the condition of being heated, the methylene blue was assigned as an indicator, then the conduction was performed with standard solution. The red cuprous oxide precipitate was formed after the reducing sugar in the sample solution reacted with potassium sodium copper tartrate. The excess reducing sugar would reduce methylene blue after all of the Cu^2+^ was used up, and the color of the solution was changed gradually into colorless from blue, which was appointed to the end of the titration. The consumption of the reducing solution could be calculated according to the change in sugar content. After the protein was removed from the samples, methylene blue was used as the indicator to titrate the calibrated alkaline copper tartrate solution. Finally, the reducing sugar amount according to the consumption volume of the sample solution was calculated.

### 4.4. The Explore of the Cell Wall Related Genes

Based on the NCBI database numbered PRJNA675306 [32], the genes involved in the development of cell wall were explored according to the KEGG and GO annotations. All of the genes related to cell wall were then classified according to the putative functions, and the number of the characteristic genes were analyzed. The full coding sequences of the putative genes were obtained, which were subsequently translated to the protein sequences by Editseq software. The functional domains were acquired through the online software SMART (V 9) (http://smart.embl-heidelberg.de/smart/set_mode.cgi?NORMAL=1 (26 October 2020).

### 4.5. Real-Time Quantitative Reverse Transcriptase PCR

In order to analyze the expression characteristics of the cell wall-related genes and the internal control gene simultaneously in response to SAHL stress treatments, the total RNA isolated from the time-course samples was used to synthesize the first-strand complementary DNA (cDNA) with PrimeScript TM II 1st Strand cDNA Synthesis Kit (No: 6210A, TaKaRa Bio Inc., Kusatsu, Japan). A total of 0.5 μg RNA in a 20 μL reaction to cDNA by the oligo dT primers for further qRT-PCR analysis. qRT-PCR was carried out using gene-specific primer pairs listed in Additional file S1. In a total volume of 20 µL, containing 2 µL of cDNA, 10 µL SYBR mix, 0.5 µL gene-specific primers and 7.5 µL ddH_2_O, the qRT-PCR was conducted using SYBR Premix Ex Taq II Kit (No: RR420A, TaKaRa Bio Inc., Kusatsu, Japan) with a Roche Light Cycler 480 system. The PCR procedures were followed as: an initial denaturation step at 95 °C for 20 s, then 40 cycles of denaturation at 95 °C for 5 s, and annealing and extension at 60 °C for 15 s. Expression levels were normalized using the Actin transcript level [48,49,50]. The qRT-PCR was performed with four independent biologicals and two technical replicates on a ABI QuantStudio™6 Flex System (No: ABI QuantStudio6Flex, Applied Biosystems, Waltham, MA, USA) using an SYBR Green based PCR assay [48]. LinRegPCR program was employed to determine the PCR efficiency for each sample, and primer efficiency (PE) was calculated by the mean of efficiency values obtained from the individual samples [51,52]. Expression levels of tested genes were determined and calculated by 2^−∆∆Ct^ as previously described method [53].

### 4.6. Statistical Analysis

Experiments were conducted with biological triplicates from the separate microalgal cultures in this study except the transcriptome analysis. Data in the figures and tables were shown as the average of triplicates with standard errors. One-way ANOVA (SPSS 19.0) with T-test was performed for statistically analysis, and *p*-values of <0.05 were considered as statistically significant.

## 5. Conclusions

In this study, the genes involved in the formation of cellulose and mannose were explored on the base of transcriptomic data, and the results of characterized expression analysis combined with the change of components revealed that when the algal cells exposed to the tress of SAHL, the putative genes involved in the formation of cellulose were upregulated to form cellulose to provide the rigid support. In addition, the cellulose hydrolysis to provide sources of mannose synthesis. The expression of mannose genes were sharply increased to regulate the formation of mannose to protect the algal cells.

## Figures and Tables

**Table 1:** Effects of SAHL treatments on the measurements of *H. pluvialis*.

Measurements	Control (0)	SAHL_1	SAHL_6	SAHL_12	SAHL_24	SAHL_48
MC	180.13± 2.12 ^d^	234.96± 1.92 ^c^	242.14 ± 1.66 ^c^	262.89 ± 2.88 ^b^	274.16 ± 3.46 ^b^	293.89 ± 1.58 ^a^
CC	300.16 ± 2.74 ^d^	352.99 ± 4.18 ^c^	366.51 ± 4.18 ^b^	372.01 ± 6.73 ^b^	369.47 ± 3.64 ^b^	388.49 ± 4.67 ^a^
FC	0.83 ± 0.01 ^d^	1.05 ± 0.01 ^c^	1.07 ± 0.02 ^c^	1.12 ± 0.01 ^c^	1.20 ± 0.01 ^b^	1.33 ± 0.01 ^a^
GC	0.76 ± 0.01 ^d^	0.99 ± 0.01 ^c^	1.01 ± 0.01 ^c^	1.06 ± 0.01 ^b^	1.10 ± 0.01 ^b^	1.16 ± 0.01 ^a^

Data are given as means ± S.D., *n* = 3. MC is mannose content (mg·g^−1^), CC is cellulose content (ng·g^−1^), FC is fructose content (mg·g^−1^), GC is glucose content (mg·g^−1^); ^a,b,c,d^ indicate significant differences (*p* < 0.05) among treatments, and any of the same lowercase letters or letter said the difference was not significant (*p* > 0.05).

**Table 2:** The genes involved in both the cellulose and mannose development pathways and their expression level of FPKM in *H. pluvialis* in this study.

Gene_id	COG_Functional Categories	Pfam Functional Domain	Control	SAHL_1	SAHL_6	SAHL_12	SAHL_24	SAHL_48
Ch_GLEAN_10012200	Cell wall/membrane/envelope biogenesis	O-Glycosyl hydrolases	1.15	16.96	2.41	24.55	13.52	12.32
Ch_GLEAN_10011277	Cell wall/membrane/envelope biogenesis	O-Glycosyl hydrolases	3.11	3.69	3.32	3.74	2.31	3.16
MSTRG.46576	Cell wall/membrane/envelope biogenesis	GDP-D-mannose pyrophosphorylase	3.46	6.09	5.31	11.02	25.98	149.19
MSTRG.2363	Cell wall/membrane/envelope biogenesis	Glucose/GDP mannose dehydrogenase	7.52	11.54	7.79	11.96	8.85	7.84
MSTRG.30653	Cell wall/membrane/envelope biogenesis	UDP-arabinopyranose mutases	2.78	4.06	4.35	4.81	7.22	6.24
MSTRG.51753	Carbohydrate transport and metabolism	Mannitol dehydrogenase	2.7	2.8	3.66	3.62	4.16	4.28
MSTRG.6412	Carbohydrate transport and metabolism	Glucose-6-phosphate 1-epimerase	4.13	3.91	5.06	4.82	4.95	7.05

**Table 3:** Correlations between expression of carotenogenic and fatty acid biosynthesis genes (cofactors, Pearson Correlation in SPSS 19.0).

Genes	Ch_GLEAN_10012200	Ch_GLEAN_10011277	MSTRG.46576	MSTRG.2363	MSTRG.30653	MSTRG.51753	MSTRG.6412
MC	0.552	−0.176	0.878 *	0.155	0.885 *	0.893 *	0.891 *
CC	0.862 *	−0.002	0.542	0.818 *	0.881 *	0.826 *	0.679
FC	0.465	−0.210	0.747	0.052	0.879 *	0.890 *	0.858 *
GC	0.558	−0.130	0.596	0.199	0.863 *	0.860 *	0.693

* Indicated that there was a significant correlation between the expression genes at *p* < 0.05 level.

## Data Availability

The datasets used and analyzed during the current study are available from the corresponding author on reasonable request.
